# Supplementary material for: Heparan sulfate assists SARS-CoV-2 in cell entry and can be targeted by approved drugs in vitro
Source: bioRxiv. 2020 Sep 18:2020.07.14.202549. Originally published 2020 Jul 14. Preprint. [Version 2] doi: 10.1101/2020.07.14.202549 (PMC7373127; doi:10.1101/2020.07.14.202549)
Supplement: 1 [file NIHPP2020.07.14.202549-supplement-1.pdf]

bioRxiv preprint doi: https://doi.org/10.1101/2020.07.14.202549. this version posted September 18, 2020. The copyright holder for this preprint (which was not certified by peer review) is the author/funder. This article is a US Government work. It is not subject to copyright under 17 USC 105 and is also made available for use under a CC0 license.

Supplementary Table S1

Reagents and Materials	Source	Catalogue No.
pHrodo™ Red, succinimidyl ester (pHrodo™ Red, SE)	ThermoFisher Scientific	Cat# P36600
Banoxantrone dihydrochloride	Sigma	Cat# SML1854
Heparin sodium salt from porcine intestinal mucosa	Sigma	Cat# H3393
Heparan sulfate sodium salt	Biosynth Carbosynth	YH30121
Chondroitin sulfate sodium salt	Biosynth Carbosynth	YH04273
SARS-CoV-S and SARS-CoV2-S Pseudotyped particles	Codex Biosolutions (Gaithersburg, MD)	Contracted custom production
ATPLite	PerkinElmer	Cat# 6016736
Bright-Glo Luciferase kit	Promega	Cat# E2620
CellTiter-Glo cell viability kit	Promega	Cat# G7572
iScript™ Reverse Transcription Supermix for RT-qPCR	BioRad	Cat# 1708840
SsoAdvanced Universal SYBR Green Supermix	BioRad	Cat# 1725271
Imaging chamber	iBidi	Cat# 80426
TriPure reagent	Sigma	Cat# 11667157001
RNeasy MinElute Cleanup Kit	Qiagen	Cat# 74024
Latrunculin A	TOCRIS	Cat# 3973
NCGC00015693-09	Microsource	01503278
NCGC00015889-11	Microsource	01505622
NCGC00024857-02	BIOMOL	AC-304
NCGC00094226-07	SigmaAldrich	Lopac-P-0453
NCGC00094226-13	Selleck	S3026
NCGC00095196-06	Microsource	02300009
NCGC00164631-05	Sequoia	SRP01785s
NCGC00186034-01	SigmaAldrich	Lopac-K-1015
NCGC00186034-03	SIGMA	K1015
NCGC00249050-02	GVK	FFS-12-73-NCI-3078
Spike S1_S2	Sino Biological	40589-V27B-B
pLV-mCherry	A gift from Pantelis Tsoulfas (Addgene #36084)	
pcDNA3.1-SARS-CoV2-Spike	BEI resources	NR-52420
SARS-CoV-2 S antibody	GeneTex	Cat# GTX632604
